# Evaluation of the Pattern of EPIYA Motifs in the *Helicobacter pylori cagA* Gene of Patients with Gastritis and Gastric Adenocarcinoma from the Brazilian Amazon Region

**DOI:** 10.1155/2014/418063

**Published:** 2014-04-24

**Authors:** Adenielson Vilar e Silva, Mario Ribeiro da Silva Junior, Ruth Maria Dias Ferreira Vinagre, Kemper Nunes Santos, Renata Aparecida Andrade da Costa, Amanda Alves Fecury, Juarez Antônio Simões Quaresma, Luisa Caricio Martins

**Affiliations:** ^1^Laboratório de Patologia Clínica, Núcleo de Medicina Tropical, Universidade Federal do Pará, Avenida Generalissimo Deodoro No. 92, Umarizal, 66095-360 Belém, PA, Brazil; ^2^Núcleo de Medicina Tropical, Laboratório de Patologia Clínica das Doênças Tropicais, Universidade Federal do Pará, Belém, PA, Brazil; ^3^Departamento de Gastroenterologia, Hospital Ophir Loyola, Belém, PA, Brazil

## Abstract

The *Helicobacter pylori* is associated with the development of different diseases. The clinical outcome of infection may be associated with the *cagA* bacterial genotype. The aim of this study was to determine the EPIYA patterns of strains isolated from patients with gastritis and gastric adenocarcinoma and correlate these patterns with the histopathological features. Gastric biopsy samples were selected from 384 patients infected with *H. pylori*, including 194 with chronic gastritis and 190 with gastric adenocarcinoma. The presence of the *cagA* gene and the EPIYA motif was determined by PCR. The *cagA* gene was more prevalent in patients with gastric cancer and was associated with a higher degree of inflammation, neutrophil activity, and development of intestinal metaplasia. The number of EPIYA-C repeats showed a significant association with an increased risk of gastric carcinoma (OR = 3.79, 95% CI = 1.92–7.46, and *P* = 0.002). A larger number of EPIYA-C motifs were also associated with intestinal metaplasia. In the present study, infection with *H. pylori* strains harboring more than one EPIYA-C motif in the *cagA* gene was associated with the development of intestinal metaplasia and gastric adenocarcinoma but not with neutrophil activity or degree of inflammation.

## 1. Introduction

*Helicobacter pylori* is a spiral Gram-negative bacterium that infects the stomach and causes chronic gastritis. In addition, the bacterium plays an important role in the pathogenesis of gastroduodenal ulcer and gastric carcinoma [1]. The diverse clinical outcomes of* H. pylori* infection depend on factors such as bacterial virulence, host susceptibility, and environmental factors [2].

Protein-associated gene A (CagA) is an important virulence factor of* H. pylori* that is found in 70 to 80% of strains isolated in Brazilian cities and is associated with the development of peptic ulcers and gastric carcinoma [3, 4]. This protein is encoded by the* cagA* gene, which is located on the cag pathogenicity island (cag-PAI). After adhesion of* cagA*-positive* H. pylori* strains to the gastric epithelium, the CagA protein is injected directly into the host cell through a type IV secretion system encoded by the cag-PAI. Inside the epithelial cell, CagA is phosphorylated in its carboxy-terminal region. This region is highly variable and contains a polymorphic pattern of Glu-Pro-Ile-Tyr-Ala amino acid repeats (EPIYA motif) [5, 6]. Four types of EPIYA segments have been described (EPIYA-A, -B, -C, and -D) [7, 8]. CagA proteins always possess the EPIYA-A and EPIYA-B sites, but some proteins also contain one or more repeats of the EPIYA-C site. This pattern is found normally in strains circulating in Western countries such as Europe, North America, and Australia (western CagA), whereas the presence of EPIYA-A and EPIYA-B sites, followed by the EPIYA-D site, has been described for* H. pylori* strains isolated in Asian countries [6, 8, 9].

The EPIYA-C and -D motifs are the main sites for phosphorylation of CagA. Phosphorylated CagA forms a physical complex with SHP-2 phosphatase and triggers abnormal cellular signals that lead to the deregulation of cell growth, cell-to-cell contact, and cell migration, elongation of epithelial cells, and increased epithelial cell turnover, increasing the risk of acquiring precancerous genetic changes. The presence of the EPIYA-D motif or of multiple EPIYA-C repeats is associated with increased SHP-2 phosphatase activity induced by CagA [10, 11]. In Western countries, infection with strains carrying EPIYA-C repeats has been shown to predispose to the development of precancerous lesions and gastric cancer [8, 11].

Studies, conducted in Pará state, have demonstrated a high frequency of the* cagA* gene among circulating strains. In addition, CagA was found to be associated with peptic ulcers and gastric cancer [3, 12]. However there are no studies of polymorphism of CagA in bacterial strains isolated in the Amazon region. And even in Brazil such studies are scarce [13, 14].

The objective of the present study was to determine the prevalence of variants of the 3′-region of the* cagA* gene among strains isolated from patients with chronic gastritis and gastric carcinoma and to investigate the association between these variants and histopathological features of the diseases.

## 2. Materials and Methods

### 2.1. Patients

Participated in the study were a total of 384 patients infected with* H. pylori* (222 men and 162 women) seen between May 2010 and June 2011 at Hospital Ophir Loyola, Belém, Pará, Brazil. All subjects included in the study were of the same socioeconomic level, had similar cultural habits, were born in the State of Pará, and had the same ethnic background (approximately 50% Portuguese, 40% Amerindian, and 10% African) [15].

The study was approved by the Ethics Committee of the Tropical Medicine Center, Belém, Pará, Brazil. All patients gave their informed consent to participate in the study.

During endoscopy, two biopsies of the area adjacent to the lesion (perilesion) were obtained from patients with a suspicion of carcinoma for histological analysis and two antral biopsy specimens were obtained for analysis by molecular methods. Four antral biopsies (two for histological analysis and two for molecular analysis) were obtained from patients with gastritis.

### 2.2. Histological Analysis

The two antral biopsy specimens (one from the greater curvature and one from the incisura angularis) were fixed in 10% buffered formalin, embedded in paraffin, cut into sequential 0.4 *μ*m sections, and stained with hematoxylin and eosin. The biopsy specimens were analyzed by an experienced pathologist who was unaware of the clinical data of each patient. Histopathological parameters were graded from 0–3 (corresponding to absent, mild, moderate, and intense) using the criteria of the updated Sydney classification system [16] for chronic inflammation, polymorphonuclear activity, and intestinal metaplasia. Gastric carcinoma cases were classified using the Lauren classification [17].

### 2.3. Extraction of* H. pylori* Genomic DNA

Genomic DNA was extracted from the antral biopsy specimens using the PureLink Genomic DNA Mini kit (Invitrogen, São Paulo, Brazil).

### 2.4. Detection of* H. pylori*

PCR amplification for the detection of* H. pylori* DNA in gastric mucosa was performed as previously described [18]. Briefly, one set of primers (p1-F and p2-R) that amplify a fragment of 298 bp of the 26-kDa antigen gene present in all* H. pylori* strains was used for detection of the bacterium. The study included samples from patients in which bacterial DNA was isolated.

### 2.5. Amplification of the Constant Region of the* cagA* Gene

The constant region of the* cagA* gene was analyzed in samples positive for* H. pylori*. All patients positive for this region were then submitted to investigation of variable region (EPIYA) polymorphisms. The constant region of the* cagA* gene was amplified by the polymerase chain reaction (PCR) using the CagA/ConF 5′-GTGCCTGCTAGTTTGTCAGCG-3′ and CagA/ConR 5′ TTGGAAACCACCTTTTGTATTAGC-3′ primers [14]. Negative and positive controls were included in all reactions. The PCR products were separated by electrophoresis on 2% agarose gel that was stained with ethidium bromide and visualized under a UV transilluminator.

### 2.6. Amplification of the 3′-Variable Region of the* cagA* Gene and Determination of the EPIYA Pattern

The following primers described by Yamaoka et al. [19] were used for amplification of the 3′-variable region of the* cagA* gene: CAG1: 5′-ACCCTAGTCGGTAATGGGTTA-3′ and CAG2: 5′-GTAATTGTCTAGTTTCGC-3′. The reaction consisted of 0.5 mM of each primer, 1X PCR buffer, 1.5 mM MgCl2, sterile water, 0.2 mM of each deoxynucleotide, 1.25 *μ*L Taq DNA polymerase (Invitrogen), and 2 *μ*L DNA in a final volume of 25 *μ*L. The amplification conditions were initial denaturation at 95°C for 2 min, followed by 35 cycles of denaturation at 95°C for 1 min, annealing at 56°C, and extension at 72°C for 1 min, with a final extension at 72°C for 10 min. PCR was carried out in a thermocycler GeneAmp PCR system 9700 (Applied Biosystems). Products of 500 to 850 bp were obtained depending on the type and number of repeats of the EPIYA-C motif in the* cagA* gene. The PCR products were separated by electrophoresis on 2% agarose gel that was stained with ethidium bromide and visualized under a UV transilluminator. Positive controls of the different patterns of the EPIYA motif were used to confirm the PCR results. This methodology also allows the detection of mixed infection.

### 2.7. Sequencing of the 3′-Variable Region of* cagA*

PCR products were purified with the Wizard SV Gel and PCR Clean-Up System (Promega, Madison, MI) according to the manufacturer's recommendations. Purified products were sequenced using a BigDye Terminator v3.1 Cycle Sequencing Kit in an ABI 3130 Genetic Analyzer (Applied Biosystems, Foster City, CA). The sequences obtained were aligned using the CAP3 Sequence Assembly Program (available from: http://pbil.univ-lyon1.fr/cap3.php/). After alignment, nucleotide sequences were transformed into aminoacid sequences using the Blastx program (available from: http://blast.ncbi.nlm.nih.gov/Blast.cgi/) and compared to sequences deposited into the GenBank (http://www.ncbi.nlm.nih.gov/Genbank/).

### 2.8. Statistical Analysis

Odds ratios were calculated to evaluate the association of the* cagA* gene and EPIYA motifs with gastric diseases and histological parameters and the Mann Whitney *U* test was used to compare frequencies, adopting a level of significance of 95%. Statistical analysis was performed using the Biostat 5.0 program [20].

## 3. Results

A total of 384 patients with gastric symptoms and infected by* H.pylori* participated in the study. According to the histological report, 50.52% (194/384) of the patients had chronic gastritis and 49.48% (190/384) had gastric adenocarcinoma, including 32.11% (61/190) with the diffuse type and 67.89% (129/190) with the intestinal type.

Age ranged from 18 to 63 years (mean: 37.2 years) for patients with chronic gastritis and from 27 to 90 years (mean: 59.9 years) for patients with gastric cancer. With respect to gender, there was a predominance of men in the two groups, with a frequency of 63.16% (120/190) in the cancer group and of 52.58% (102/194) in the gastritis group.

Investigation of the* cagA* gene in gastric biopsies showed that 256/384 (66.7%) of the patients harbored strains carrying this gene. This frequency was higher among patients with adenocarcinoma 159/190 (82.1%) than among those with gastritis 100/194 (51.5%) (OR = 4.31, 95%IC (2.70–6.87), *P* < 0.01).

To examine the association between different patterns of EPIYA with gastric diseases and histopathological data was used only samples monoinfected.

### 3.1. Determination of the EPIYA Pattern of the* cagA* Gene and Its Association with Gastric Diseases

The distribution of the EPIYA genotypes is shown in Table 1. The sequencing confirmed the results obtained by PCR and was not found EPIYA-D sites in the* H. pylori* strains studied.

Analysis of the different EPIYA motifs in the 3′-region of the* cagA* gene revealed that 58% (150/256) of the patients infected with* cagA*-positive* H. pylori* harbored strains with only one* cagA* EPIYA genotype (monoinfected) and 42% (106/256) presented mixed infection with strains carrying different* cagA* EPIYA genotypes (Table 1). The frequency of mixed infection was higher among patients with adenocarcinoma compared with those with gastritis (OR = 2.99, 95% IC (1.73–5.13), *P* < 0.01).

The analysis of the distribution of the EPIYA patterns in monoinfected patients showed that colonization by* H. pylori* CagA-positive with two or three EPIYA C motifs was more prevalent among patients with gastric adenocarcinoma (OR = 3.78, 95% IC (1.92–7.46), *P* = 0.002).

### 3.2. Association between Presence of the* cagA* Gene and Histopathological Findings

When the histopathological findings of the patients colonized by CagA-positive strains (256/384) with those harboring CagA-negative strains (128/384) were compared, a higher degree of gastric inflammation, neutrophil activity, and intestinal metaplasia was observed in the former (Table 2).

### 3.3. Association between the Numbers of EPIYA-C Segments and Histopathological Findings

The increased number of EPIYA-C segments was associated with the presence of intestinal metaplasia (Table 3) but not with the other histological parameters such as degree of inflammation and neutrophil activity (Table 4).

## 4. Discussion

The CagA protein is an important* H. pylori* virulence marker that is associated with diseases such as peptic ulcer and gastric carcinoma in Western countries [21, 22]. Studies conducted in different Brazilian states, including the State of Pará, have demonstrated a high prevalence of strains carrying the* cagA* gene in the Brazilian population, as well as an association of these strains with peptic ulcer disease and gastric carcinoma [3, 12, 23]. Similar results were observed in the present study in which the prevalence of strains carrying the* cagA* gene was higher among patients with gastric adenocarcinoma than among those with gastritis.

In addition to the presence of the* cagA* gene, the type of EPIYA motif in the carboxy-terminal region of the gene has recently been shown to influence the biological activity of CagA and the aggressiveness of* H. pylori* strains [11, 24]. A study conducted in Western countries demonstrated that the presence of bacterial strains with multiple repeats of the EPIYA-C motif predisposes to precancerous lesions and gastric cancer [24].

All patients in this study were natives of the state of Pará, and based on the study of Santos et al., 1999, the population of this state has an ethnic background of approximately 50% Portuguese, 40% Amerindian, and 10% African. In Brazil, a continental country, there are few studies on this subject, this study being the first to evaluate the pattern of CagA EPIYA segments in the North region of the country.

In the present study strains with two or three repeats of the EPIYA-C motif were more frequent in patients with gastric adenocarcinoma. The risk of gastric cancer was approximately 4-fold higher among patients infected with* cagA*-positive strains carrying two or three EPIYA-C motifs compared to patients infected with* cagA*-positive strains carrying no or only one EPIYA-C motif. Furthermore, a higher frequency of colonization with mixed strains harboring different types of EPIYA motifs was observed in patients with adenocarcinoma. Similar results have been reported by Batista et al. [13] who studied patients from Minas Gerais, Brazil.

Although several studies have demonstrated an association between infection with* H. pylori* strains harboring two or three EPIYA-C motifs and gastric cancer, no association could be established between the number of EPIYA-C repeats and increased gastric inflammation [24, 25].

In the present study a positive association was observed between* cagA* status and neutrophil activity, degree of inflammation, and intestinal metaplasia. However, there was no association between the number of EPIYA-C repeats and the degree of lymphocyte or neutrophil infiltration.

CagA has been associated with increased gastric inflammation characterized by high-grade leukocyte infiltration, which is a long-term risk factor for carcinogenesis since the intense oxidative process triggered by the disintegration of cells in the gastric mucosa releases potentially mutagenic substances [26, 27]. Some studies suggest that the number of EPIYA-C repeats is not associated with proinflammatory cytokine production but rather increases the binding to SHP-2, with consequent morphological changes in the cell that induce mucosal damage [25, 28].

## 5. Conclusions

*H. pylori* strains harboring two or three EPIYA-C motifs, which are a risk factor for gastric cancer, predominated in patients with adenocarcinoma and were associated with the development of intestinal metaplasia in the gastric mucosa.

## Figures and Tables

**Table 1:** Distribution of *Helicobacter pylori cagA *EPIYA genotypes in patients with chronic gastritis and gastric carcinoma.

	EPIYA pattern	Gastritis *n* (%)	Cancer *n* (%)
Monoinfection	EPIYA-AB	4 (5.40)	4 (5.26)
	EPIYA-ABC	42 (56.76)	19 (25)
	EPIYA-ABCC	26 (35.14)	25 (32.90)
	EPIYA-ABCCC	2 (2.70)	28 (36.84)

Mixed infection	EPIYA-ABC/ABCC	20 (76.92)	33 (41.25)
	EPIYA-ABC/ABCCC	—	2 (2.5)
	EPIYA-ABCC/ABCCC	2 (7.7)	14 (17.5)
	EPIYA-ABC/ABCC/ABCCC	4 (15.38)	31 (38.75)

**Table 2:** Association between the presence or absence of the *cagA *gene and the histopathological findings of the study participants.

Histopathological finding	*cagA* gene		OR (95% CI)	*P*
	Positive (%)	Negative (%)		
Degree of inflammation				
Mild	39 (15.23)	50 (39.06)	3.57 (2.18–5.84)	0.0001
Moderate to intense	217 (84.77)	78 (60.94)		
Neutrophil activity				
Mild	51 (20)	94 (73.44)	11.11 (5.48–22.30)	0.0001
Moderate to intense	205 (80)	34 (26.56)		
Metaplasia				
Mild	39 (15.23)	6 (4.69)	3.65 (1.50–8.88)	0.004
Moderate to intense	217 (84.77)	122 (95.31)		

**Table 3:** Association between EPIYA polymorphisms and the presence or absence of metaplasia in monoinfected patients with chronic gastritis and gastric carcinoma.

	Gastritis				Cancer			
	Metaplasia		OR (95% CI)	*P*	Metaplasia	
	Present	Absent			Present	Absent
EPIYA (AB or ABC)	1	46	13.14 (1.49–116.15)	0.01	2	20	5.00 (1.0509–237.89)	0.05
EPIYA (ABCC or ABCCCC)	6	21			18	36		

Total	7	67			20	56.		

**Table 4:** Association between EPIYA polymorphisms in the *cagA* gene and degree of inflammation and neutrophil activity in monoinfected patients.

	Degree of inflammation			Neutrophil activity		
	EPIYA pattern		*U* (*p*)	EPIYA pattern	
	AB or ABC	ABCC or ABCCCC		AB or ABC
Gastritis	2 (1–3)	2 (1–3)	217.50 (0.52)	2 (1–3)	2 (1–3)	280.50 (0.41)
Cancer	2 (1–3)	2 (1–3)	592.50 (0.88)	2 (1–3)	2 (1–3)	569.00 (0.67)

Mild (1); moderate (2); intense (3).
